# Children’s Mapping between Non-Symbolic and Symbolic Numerical Magnitudes and Its Association with Timed and Untimed Tests of Mathematics Achievement

**DOI:** 10.1371/journal.pone.0093565

**Published:** 2014-04-03

**Authors:** Carmen Brankaer, Pol Ghesquière, Bert De Smedt

**Affiliations:** Faculty of Psychology and Educational Sciences, KU Leuven, Leuven, Belgium; Langeveld Institute, Utrecht University, Netherlands

## Abstract

The ability to map between non-symbolic numerical magnitudes and Arabic numerals has been put forward as a key factor in children’s mathematical development. This mapping ability has been mainly examined indirectly by looking at children’s performance on a symbolic magnitude comparison task. The present study investigated mapping in a more direct way by using a task in which children had to choose which of two choice quantities (Arabic digits or dot arrays) matched the target quantity (dot array or Arabic digit), thereby focusing on small quantities ranging from 1 to 9. We aimed to determine the development of mapping over time and its relation to mathematics achievement. Participants were 36 first graders (*M = *6 years 8 months) and 46 third graders (*M* = 8 years 8 months) who all completed mapping tasks, symbolic and non-symbolic magnitude comparison tasks and standardized timed and untimed tests of mathematics achievement. Findings revealed that children are able to map between non-symbolic and symbolic representations and that this mapping ability develops over time. Moreover, we found that children’s mapping ability is related to timed and untimed measures of mathematics achievement, over and above the variance accounted for by their numerical magnitude comparison skills.

## Introduction

Numerical magnitude processing, and more specifically the ability to map between non-symbolic and symbolic magnitude representations, is proposed to play an important role in the development of mathematics [1]–[3]. Most studies examined the association between this mapping ability and mathematics achievement indirectly by looking at children’s performance on a classic symbolic magnitude comparison task [4], [5], yet Mundy and Gilmore [3] were able to examine mapping more directly by using a novel task in which children had to choose which of two quantities (dot arrays or Arabic digits) matched a target quantity (Arabic digit or dot array), thereby focusing on quantities larger than 20. They found that children’s mapping ability increased between 6 and 8 years of age and that this ability was related to children’s performance on an untimed test of school mathematics. However, Sullivan and Barner [6] suggested that a different mapping mechanism operates for linking small vs. large non-symbolic and symbolic quantities and therefore, it remains to be determined whether the findings of Mundy and Gilmore [3] can also be observed in smaller quantities. We extended the study of Mundy and Gilmore [3] by focusing, for the first time, on the mapping of small quantities ranging from 1 to 9. We also investigated the association between this mapping ability and mathematics performance by including both timed and untimed standardized tests of mathematics achievement. In the remainder of this introduction, we first focus on numerical magnitude processing and its relation with mathematics. Secondly, we concentrate on mapping between non-symbolic and symbolic magnitude representations and finally, we present the goals and design of the current study.

### 1.1 Numerical Magnitude Processing

The ability to understand and process numerical magnitude information, commonly referred to as “number sense” [7], emerges very early in development, as infants [8] and kindergarteners [9] are able to compare and add sets of non-symbolic objects or dots. It has been suggested that over the course of development, children learn to link these non-symbolic magnitude representations with number words and Arabic digits [10], i.e. they learn to map the system for non-symbolic representations with a new, and more precise, symbolic system to represent numerical magnitudes [3]. This ability to represent numerical magnitudes has been related to mathematics achievement (e.g., [11] for a review), cross-sectionally [4], [12] and even predictively [13]–[16] or as a retrospective prediction [17]. Furthermore, children with mathematical difficulties or dyscalculia seem to have impairments with the understanding and processing of numerical magnitudes [18]–[20] and show abnormalities in brain areas that are involved in numerical magnitude processing (see [21]).

Several studies have tried to disentangle whether children’s representation of magnitude per se or children’s access to numerical meaning from symbols is crucial for successful mathematical development. This is typically done by comparing children’s performance on symbolic (Arabic digits) and non-symbolic (dot patterns) magnitude comparison tasks (e.g., [4], [18], [22]). While the findings of these studies remain to be inconclusive (see [23] for an overview and explanation of these data), several studies pointed to the importance of the ability to access numerical magnitude information from symbols for the development of mathematical skills. For example, Holloway and Ansari [4] found that children’s symbolic but not non-symbolic skills were associated with their mathematics achievement. Related to this, Rousselle and Noël [5] as well as De Smedt and Gilmore [18] found that children with mathematical difficulties were only impaired on a symbolic but not on a non-symbolic magnitude comparison task. These findings seem to suggest that the mapping between Arabic digits and the numerical magnitudes they represent is important for mathematical development and that this mapping process seems to be altered in children with mathematical difficulties.

### 1.2 Mapping between Symbolic and Non-symbolic Magnitudes

It is important to point out that the abovementioned studies only considered numerical magnitude comparison tasks to examine children’s ability to map between symbolic and non-symbolic magnitudes, which only provide an indirect measure of mapping skills [3]. Lipton and Spelke [24] were the first to investigate this mapping ability in a more direct way by asking 5-year-old children to (1) verbally estimate the number of items on a set of cards, (2) choose one card out of two with the same number of items as the number word they were given and (3) estimate the number of items on a card after letting them know how many items another card contained. They found that children were able to map number words onto non-symbolic magnitudes as soon as they had learned the count sequence. Similarly, Barth, Starr and Sullivan [25] asked children to verbally estimate the number of items on a card. Expanding the results of Lipton and Spelke [24], Barth et al. [25] observed that even children who mastered very little of the verbal count sequence had some knowledge of how large number words map onto non-symbolic magnitudes. Further, Izard and Dehaene [26] examined mapping by asking adults to estimate the numerosity of dot arrays, both with and without calibration by a reference trial (i.e. participants were, sometimes misleadingly, told how many dots there were in a reference trial). Results showed that, although adults tended to systematically underestimate the true numerosity, they were able to map from non-symbolic to symbolic representations as long as calibration was provided. More recently, Mazzocco et al. [2] asked 14- and 15-year olds to verbally estimate the numerosity of dot arrays briefly presented. Results showed that children with dyscalculia were impaired in their mapping ability from non-symbolic to symbolic representations, even when controlling for domain-general abilities.

While the previous studies mainly focused on mapping in one direction, i.e. producing a symbolic label for a non-symbolic magnitude, Mundy and Gilmore [3] investigated mapping between non-symbolic and symbolic representations in both directions by using a task in which children were shown one target quantity (Arabic digit or dot array) and had to choose which of two alternative choice quantities (dot arrays or Arabic digits) matched the target quantity. The target quantities varied from 20 to 50. In their first experiment, they showed that 6- and 8-year-old children could map in both directions between symbolic and non-symbolic representations, although accuracy was higher when mapping from non-symbolic to symbolic representations than vice versa. They also found that children’s mapping ability increased with age. In a second experiment in 7-year-olds, Mundy and Gilmore [3] further showed that this mapping ability was significantly related to individual differences in mathematics achievement, over and above the variance that was accounted for by symbolic and non-symbolic magnitude comparison performance.

Examining these issues in healthy adults, Castronovo and Göbel [1] recently analyzed non-symbolic to symbolic mapping in adults with a numerosity perception task and symbolic to non-symbolic mapping with a numerosity production task, using target quantities ranging from 21 to 98. In the first task, participants had to estimate the number of dots in a dot array, while in the second task participants had to produce a set of dots of a quantity corresponding approximately to the Arabic digit presented. Performance on these tasks was significantly related to individual differences in mathematics achievement, indicating that better mapping abilities between non-symbolic and symbolic representations were associated with higher mathematics achievement.

Both Mundy and Gilmore [3] and Castronovo and Göbel [1] examined mapping in two directions (non-symbolic to symbolic and symbolic to non-symbolic representations), but they only focused on quantities larger than or equal to 20. However, little is known about children’s mapping ability for smaller quantities. This is particularly relevant in view of recent findings by Sullivan and Barner [6], which indicate that a different mapping mechanism operates for linking small (≤ 12) vs. large (>20) non-symbolic and symbolic quantities. More specifically, Sullivan and Barner [6] showed that adults rely on item-by-item associative mappings for small quantities, but that larger quantities become mapped onto each other on the basis of their shared ordinal structure.

Further, it is also important to note that Castronovo and Göbel [1] and Mundy and Gilmore [3] used different types of mathematics achievement tests, i.e. timed and untimed tests, respectively, to investigate the association between mapping ability and mathematics achievement. Although both types of mathematics achievement tests are related to each other, recent work has indicated that timed and untimed mathematics achievement tests are measuring genetically different aspects of math performance [27]. Therefore, it is important to include both types of achievement tests to obtain a more complete picture of children’s mathematics achievement.

### 1.3 The Present Study

Extending the existing body of data, the aim of the present study was three-fold. Firstly, we aimed to examine directly mapping between non-symbolic and symbolic representations in both directions by focusing on small number magnitudes ranging from 1 to 9. This was done because the ability to map between symbols and quantities has been highlighted as an important factor for successful mathematical development, e.g. [1], [3]. Similar to Mundy and Gilmore [3], we focused on typically developing 6- and 8-year-olds and studied their mapping ability over time. We used direct mapping tasks in which children had to choose which of two choice quantities (Arabic digits or dot arrays) matched the target quantity (dot array or Arabic digit). We hypothesized that 8-year-old children would be more accurate and faster on the mapping tasks than 6-year-olds.

Secondly, we investigated the association between children’s mapping ability and their mathematics performance by using both a timed and untimed standardized test of mathematics achievement. Extending Mundy and Gilmore [3], we also examined whether the associations between children’s performance on the mapping tasks and math tests changed over developmental time by studying this relation in two age groups, i.e. 6- and 8-year-old children.

Finally, we verified whether performance on the mapping tasks could explain individual differences in mathematical achievement over and above variance accounted for by more common measures that tap into numerical magnitude processing, such as the symbolic and non-symbolic magnitude comparison tasks.

To evaluate alternative explanations for associations between the numerical processing tasks and mathematics achievement, we administered Raven’s Standard Progressive Matrices [28] and a motor reaction time task to control for potential effects of intellectual ability and processing speed, respectively.

## Methods

### 2.1 Participants

Participants were 82 children (41 boys, 41 girls) that were selected from four Flemish primary schools who had a dominantly middle- to high-income school population. Children in the first grade (*n = *36) had a mean age of 6 years 8 months (*SD = *4 months) and children from the third grade (*n = *46) had a mean age of 8 years 8 months (*SD = *3 months). None of these children had a developmental disorder and none of them had repeated grade. All children were tested in the middle of the school year (February and March).

### 2.2 Ethics Statement

Parents of all children received an information sheet on the study and provided written informed consent for their child. Given the age of our participants, children did not sign written consent but they all gave verbal agreement before undertaking the different experiments and tasks. The study and consent procedures were approved by the ethics committee of the KU Leuven (University of Leuven).

### 2.3 Materials

#### 2.3.1 Experimental tasks

The experimental tasks were presented using the E-prime 2.0 software [29]. They were all administered using a 15-inch laptop. Children were instructed to perform both accurately and quickly. Stimuli occurred in white on a black background in Arial font. Each trial was initiated by the experimenter by means of a control key and started with a 250 ms fixation cross in the centre of the computer screen. Participants had to respond by pressing a key on a computer keyboard that was put in front of the notebook and was connected to it. The left response key, labeled with a blue sticker, was ‘d’; the right response key, labeled with a yellow sticker, was ‘k’. Each task was preceded by three practice trials to familiarize the child with the key assignments. Answers and reaction times were recorded by the laptop.

Two mapping tasks were used to more directly assess children’s mapping ability between symbols and their underlying non-symbolic representations. The task design was based on Mundy and Gilmore [3]. Different from Mundy and Gilmore, we only used small quantities that varied from 1 to 9 instead of quantities ranging from 20 to 50. The dot array stimuli in both mapping tasks were generated by means of the method of Pica, Lemer, Izard and Dehaene [30] and were controlled for non-numerical parameters, i.e. individual dot size, total occupied area, and density. This was done to reduce the likelihood that children would rely on these non-numerical cues or perceptual features to make a correct decision. Stimuli disappeared after 1000 ms, in order to avoid counting.

In the *Symbolic to Non-Symbolic Mapping Task*, an Arabic symbol was presented on the computer screen, together with two dot arrays. Children had to indicate which dot array corresponded with the Arabic symbol, by pressing the response key on the side of the correct dot array. The position of the correct answer was counterbalanced. On half of the trials, the distance between the correct and the incorrect dot array was 3, on the other half of the trials this distance was 1. For each distance, half of the trials comprised numerosities in the subitizing range (1–4) while the other half of the trials involved numerosities outside the subitizing range (5–9). This task consisted of 24 experimental trials.

In the *Non-Symbolic to Symbolic Mapping Task*, the same numerosities as in the symbolic to non-symbolic mapping task were used, but now the target quantity was a set of dots and children had to choose the corresponding Arabic digit out of two digits. Again, 24 trials were presented.

Two classic numerical magnitude comparison tasks [31] were administered. In the *Symbolic Comparison Task*, children had to indicate the numerically larger of two simultaneously presented Arabic digits, one displayed on the left and one displayed on the right side of the computer screen. Stimuli involved all combinations of the digits 1 to 9, yielding 72 trials, and remained visible until response. Children had to answer by pressing the response key on the side of the larger digit. The position of the largest digit was counterbalanced.

In the *Non-Symbolic Comparison Task*, children had to indicate the larger of two simultaneously presented dot arrays, one displayed on the left and one displayed on the right side of the computer screen. Stimuli comprised the same numerosities as in the symbolic magnitude comparison task, yielding 72 trials. The stimuli were controlled for non-numerical parameters in the same way as in the mapping tasks. Stimuli disappeared after 840 ms in order to avoid counting and children had to select the larger numerosity by pressing the response key on the side of the larger numerosity. The position of the largest numerosity was counterbalanced.

A *Motor Reaction Time Task* was included as a control for children’s response speed on the keyboard. Two figures appeared on the screen at the same time. One of them was colored white and the child had to press as soon as possible on the response key on the side of this white figure. The position of the correct answer was counterbalanced. Twenty trials were presented.

#### 2.3.2 Standardized tests

*Timed Mathematics Achievement* was assessed using a standardized paper-and-pencil achievement test for arithmetic, Tempo Test Arithmetic [32]. This test consists of 200 basic arithmetic problems that are presented in five columns, each column containing one arithmetic operation (e.g., 3+5 = ). Within each column, 40 items of increasing difficulty are presented and for each column separately, children are asked to solve as many problems as possible within a one-minute period. In this study, only the first two columns (addition and subtraction, separately presented) were administered, because the children in first grade only received instruction in addition and subtraction. In both columns, the first 15 items only include single digit problems (e.g., 3+6/9−2), while the following items also include subsequently no-borrow/no-carry and borrow/carry multi-digit problems (e.g., 5+7, 13+4/15−3, 21−9). The score on this test was the number of correctly solved problems within the time-limit (maximum = 80).

*Untimed Mathematics Achievement* was assessed using a curriculum-based standardized achievement test for mathematics from a Flemish Student Monitoring System [33]. This untimed test consists of 60 items covering number knowledge, understanding of operations, (simple) arithmetic, word problem solving, measurement and geometry. The score on this test was the number of correctly solved problems.

*Raven’s Standard Progressive Matrices* [28] was used as a measure of intellectual ability. For each child, a standardized score (*M* = 100; *SD = *15) was calculated.

### 2.4 Procedure

All participants were tested at their own school during regular school hours. The standardized tests were group-based and the experimental tasks were assessed individually in a quiet room. All experimental tasks were administered in one session and all children completed the tasks in the same order.

## Results

### 3.1 Descriptive Analyses

The reaction times used in the subsequent analyses are based on correct responses only. To control for outliers, all trials that were solved faster than 250 ms or slower than 5000 ms were excluded from the analyses. To evaluate grade differences in accuracy and reaction time on the mapping and numerical magnitude comparison tasks, we calculated for each task separately repeated measures ANOVAs with task format (Mapping: Symbolic to Non-symbolic vs. Non-symbolic to Symbolic; Numerical magnitude comparison: Symbolic vs. Non-symbolic) and numerical distance (Mapping: distance 1 and 3; Numerical magnitude comparison: distance 1–8) as within-subject factors and grade (Grade 1 vs. Grade 3) as between-subjects factor. Eta-squared was computed as a measure of effect size.

#### 3.1.1 Control tasks

Children’s accuracy on the motor reaction time task was very high (Grade 1: *M = *97.49%, *SD = *3.69; Grade 3: *M = *96.63%, *SD = *4.09). There were grade differences in children’s response speed on the keyboard, *t*(80) = 6.11, *p*<.01, *η* ^2^ = .318, indicating that children from Grade 3 (*M = *448.46, *SD = *70.30) were significantly faster than children from Grade 1 (*M = *667.31, *SD = *229.87). This grade difference was considered in subsequent analyses.

Children’s intellectual ability was within the normal range (Grade 1: *M = *110.58, *SD = *14.86; Grade 3: *M = *108.54, *SD = *12.03) and there were no differences between the two grades, *t*(80) = 0.69, *p = *.49, *η* ^2^ = .006.

#### 3.1.2 Mapping tasks

**Figure 1:**
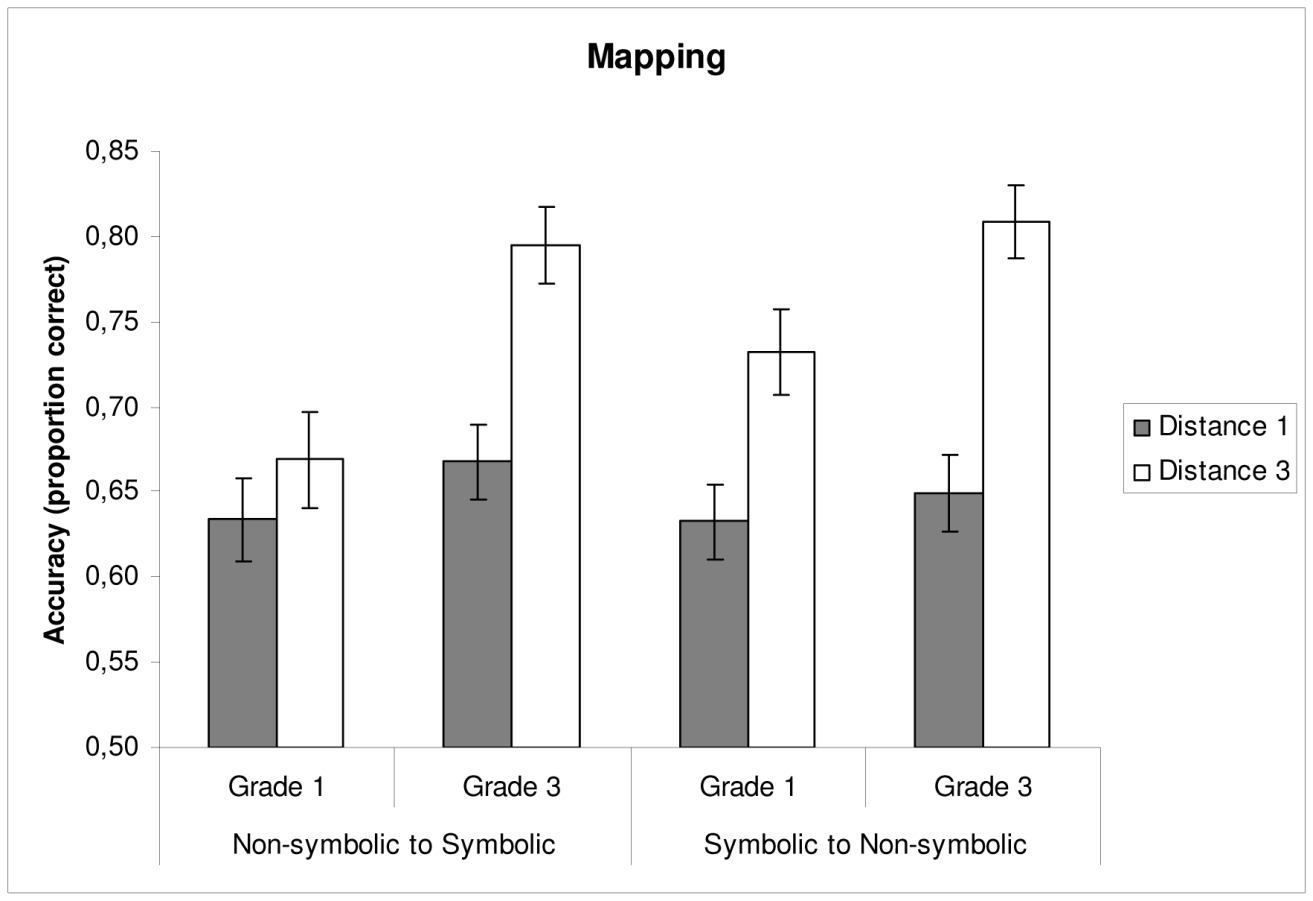
Mean accuracy on the mapping tasks as a function of grade and distance. Error bars depict 1SE of the mean.

**Figure 2:**
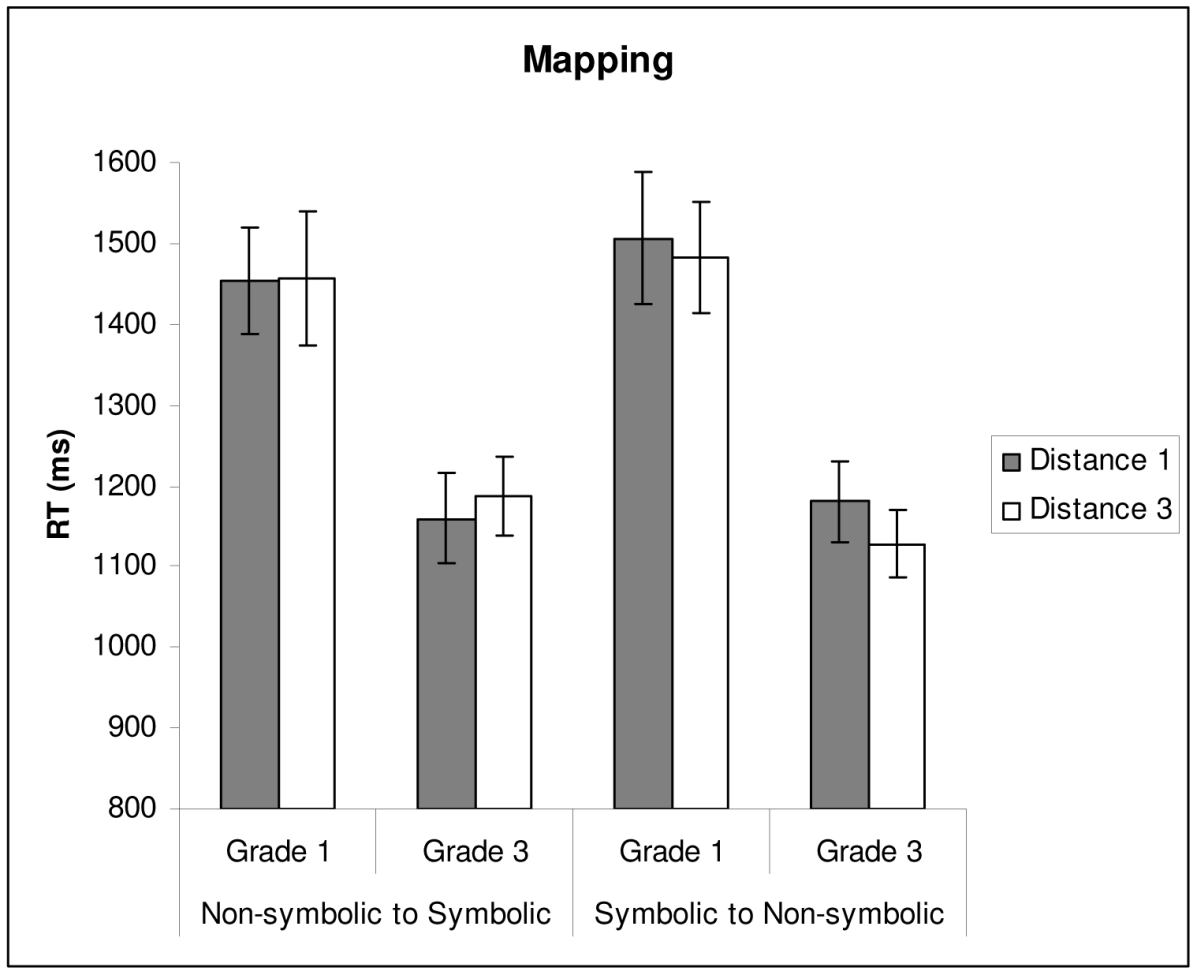
Mean reaction time (based on correct responses only) on the mapping tasks as a function of grade and distance. Error bars depict 1SE of the mean.

Descriptive statistics on the mapping tasks are presented in Figure 1 (accuracy) and Figure 2 (reaction time). With regard to accuracy, there was a main effect of Grade, *F*(1,80) = 6.63, *p* = .01, *η* ^2^ = .038, indicating that children in Grade 3 performed more accurately than children in Grade 1. There was also a main effect of Distance, *F*(1,80) = 94.56, *p<*.01, *η* ^2^ = .105, showing that problems with distance 3 between the correct and incorrect response were solved more accurately than problems with distance 1. Furthermore, a significant Grade × Distance interaction emerged, *F*(1,80) = 12.23, *p<*.01, *η* ^2^ = .014, suggesting that grade differences were larger on problems with distance 3 than on problems with distance 1. No main effect of Format, *F*(1,80) = 1.09, *p = *.30, *η* ^2^ = .002, or Grade × Format, *F*(1,80) = 1.55, *p = *.22, *η* ^2^ = .003, Format × Distance, *F*(1,80) = 2.88, *p = *.09, *η* ^2^ = .006, and Grade × Format × Distance, *F*(1,80) = 0.33, *p = *.57, *η* ^2^ = .001, interactions were found.

Turning to children’s reaction times, we only observed a main effect of Grade, *F*(1,80) = 15.52, *p<*.01, *η* ^2^ = .138: Children from Grade 3 were faster than children from Grade 1. To evaluate whether this grade difference in reaction time could be explained by differences in general reaction time, we repeated the analysis with motor reaction time as covariate. After controlling for this variable, the main effect of Grade disappeared, *F*(1,79) = 0.94, *p* = .33, *η* ^2^ = .008, indicating that general differences in response speed explained grade differences in reaction time on the mapping tasks.

#### 3.1.3 Numerical magnitude comparison

**Figure 3:**
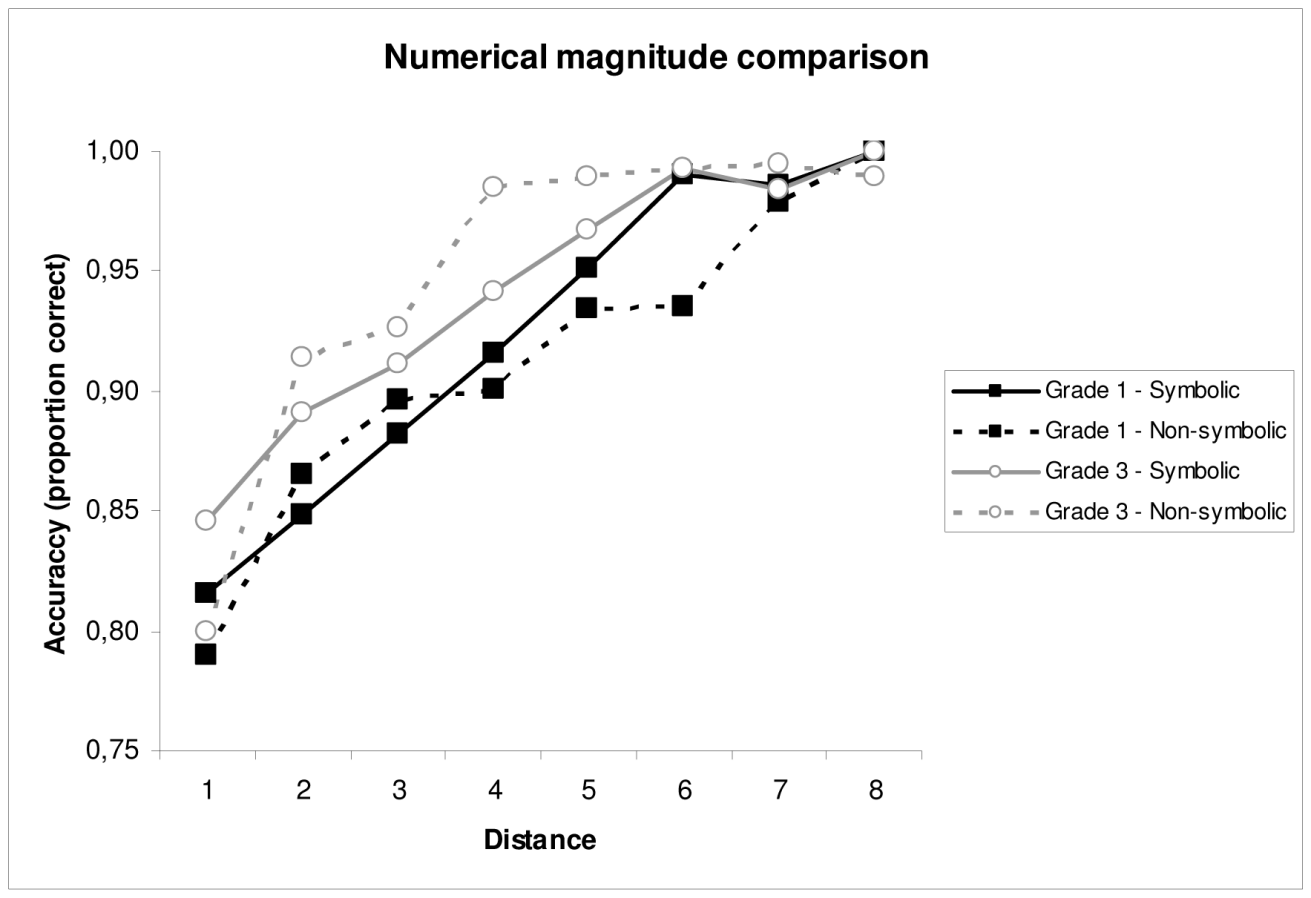
Mean accuracy on the numerical magnitude comparison tasks as a function of grade and distance. Solid lines indicate data on the symbolic magnitude comparison task (*M*_Grade 1_ = .89; *M*_Grade 3_ = .92), and dashed lines indicate data on the non-symbolic magnitude comparison task (*M*_Grade 1_ = .88; *M*_Grade 3_ = .92).

**Figure 4:**
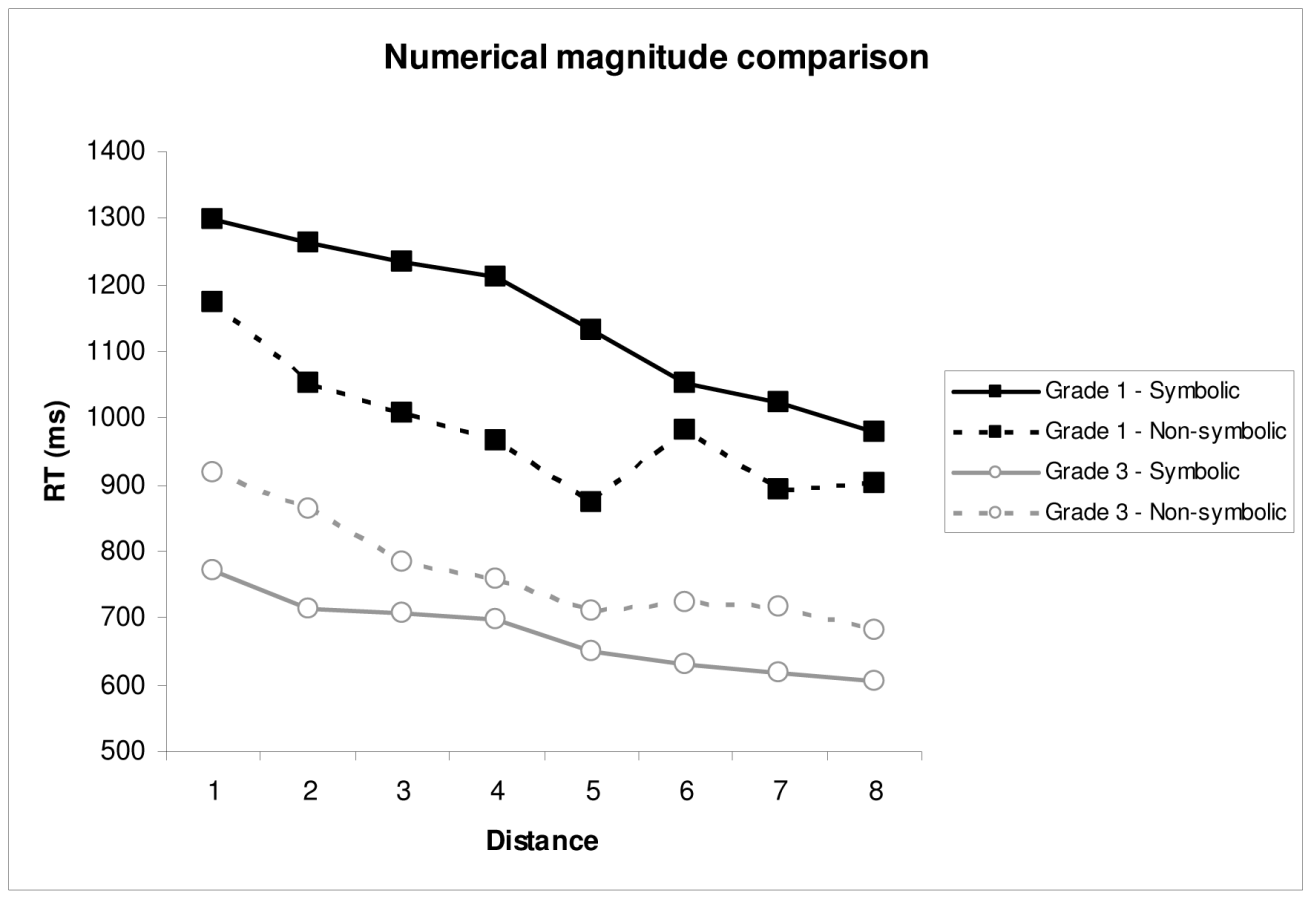
Mean reaction time (based on correct responses only) on the numerical magnitude comparison tasks as a function of grade and distance. Solid lines indicate data on the symbolic magnitude comparison task (*M*_Grade 1_ = 1199.71 ms; *M*_Grade 3_ = 698.65 ms), and dashed lines indicate data on the non-symbolic magnitude comparison task (*M*_Grade 1_ = 1013.28 ms; *M*_Grade 3_ = 799.62 ms).

Figures 3 and 4 show the descriptive statistics on the numerical magnitude comparison tasks. A main effect of Grade, *F*(1,80) = 6.52, *p* = .01, *η* ^2^ = .015, was found on children’s accuracy scores: Children from Grade 3 performed significantly more accurately than children from Grade 1. There also was a main effect of Distance, *F*(7,560) = 101.75, *p<*.01, *η* ^2^ = .291, indicating that accuracy increased when distance increased. This distance effect was larger in Grade 1 than in Grade 3, as illustrated by a significant Grade × Distance interaction, *F*(7,560) = 2.49, *p = *.02, *η* ^2^ = .007. No main effect of Format, *F*(1,80) = 0.07, *p = *.80, *η* ^2^ <.001, was found.

With regard to reaction time, there was a main effect of Grade, *F*(1,80) = 41.04, *p*<.01, *η* ^2^ = .237, indicating that children from Grade 3 were significantly faster than children from Grade 1. There was also a main effect of Distance, *F*(7,560) = 77.30, *p<*.01, *η* ^2^ = .051, showing that reaction time decreased when distance increased. A significant Grade × Distance interaction, *F*(7,560) = 3.71, *p*<.01, *η* ^2^ = .002, indicated that the distance effect was larger in Grade 1 than in Grade 3. No main effect of Format, *F*(1,80) = 2.54, *p* = .12, *η* ^2^ = .003, was found. After controlling for children’s response speed on the keyboard, the main effects of Grade, *F*(1,79) = 5.76, *p = *.02, *η* ^2^ = .024, and Distance, *F*(7,553) = 2.30, *p = *.03, *η* ^2^ = .002, remained.

#### 3.1.4 Mathematics achievement

There were grade differences on the timed mathematics achievement test, *t*(80) = 15.29, *p*<.01, *η* ^2^ = .745, indicating that children from Grade 3 (*M* = 39.28, *SD* = 6.36) solved significantly more arithmetic problems within one minute than children from Grade 1 (*M* = 18.06, *SD* = 6.08).

Because the untimed mathematics achievement test was curriculum-based, and the administered test differed in content between children from Grade 1 (*M* = 52.22, *SD* = 6.53) and children from Grade 3 (*M* = 44.85, *SD* = 7.71), no direct comparisons between both grades could be made.

### 3.2 Correlational Analyses

Firstly, Pearson partial correlation coefficients were calculated to examine the associations between the mapping tasks and the numerical magnitude comparison tasks across the two grades. Grade was included as a covariate in all analyses and children’s motor reaction time was used as a covariate in the reaction time analyses. As shown in Table 1, children’s performance on the mapping tasks was significantly correlated with their performance on the numerical magnitude comparison tasks, both for accuracy and reaction time. The same pattern of findings was obtained when the data of both grades were analyzed separately.

**Table 1:** Partial correlations between the mapping and numerical magnitude comparison tasks controlling for grade.

	1	2	3	4
**Accuracy**				
1. Mapping NS to S				
2. Mapping S to NS	.55**			
3. Comparison S	.47**	.37**		
4. Comparison NS	.40**	.36**	.45**	
**Reaction time**				
1. Mapping NS to S				
2. Mapping S to NS	.77**			
3. Comparison S	.49**	.46**		
4. Comparison NS	.81**	.65**	.50**	

Motor reaction time was additionally included as a covariate in the reaction time analyses. NS = non-symbolic; S = symbolic. *p<.05; **p<.01.

Secondly, the associations between the numerical processing tasks and the mathematics achievement tests were investigated by using Pearson correlation coefficients (see Table 2). This was done for Grade 1 and Grade 3 separately because an interaction between Grade and the non-symbolic to symbolic mapping task emerged when studying the relation between this mapping task and the untimed mathematics achievement test, *F*(1,78) = 5.88, *p* = .02, *η* ^2^ = .054. As the direction of mapping did not influence children’s performance on the mapping tasks and given the high correlations between the non-symbolic to symbolic and symbolic to non-symbolic mapping tasks, we decided to combine data of both mapping tasks by averaging the accuracies of the two mapping tasks into one accuracy score and by averaging the reaction time data of the two mapping tasks into one index for reaction time.

**Table 2:** Correlations between the experimental tasks and mathematics achievement.

	Grade 1		Grade 3	
	Timed	Untimed	Timed	Untimed
**Accuracy**				
Mapping	.19	.42*	.31*	−.04
Comparison S	.22	.11	.13	.10
Comparison NS	.36*	.15	.14	−.16
**Reaction time**				
Mapping	−.13	−.03	−.02	−.17
Comparison S	−.42*	−.44**	−.35*	−.26
Comparison NS	−.14	.02	−.17	−.20

Timed = timed mathematics achievement; Untimed = untimed mathematics achievement; NS = non-symbolic; S = symbolic. *p<.05; **p<.01.

**Figure 5:**
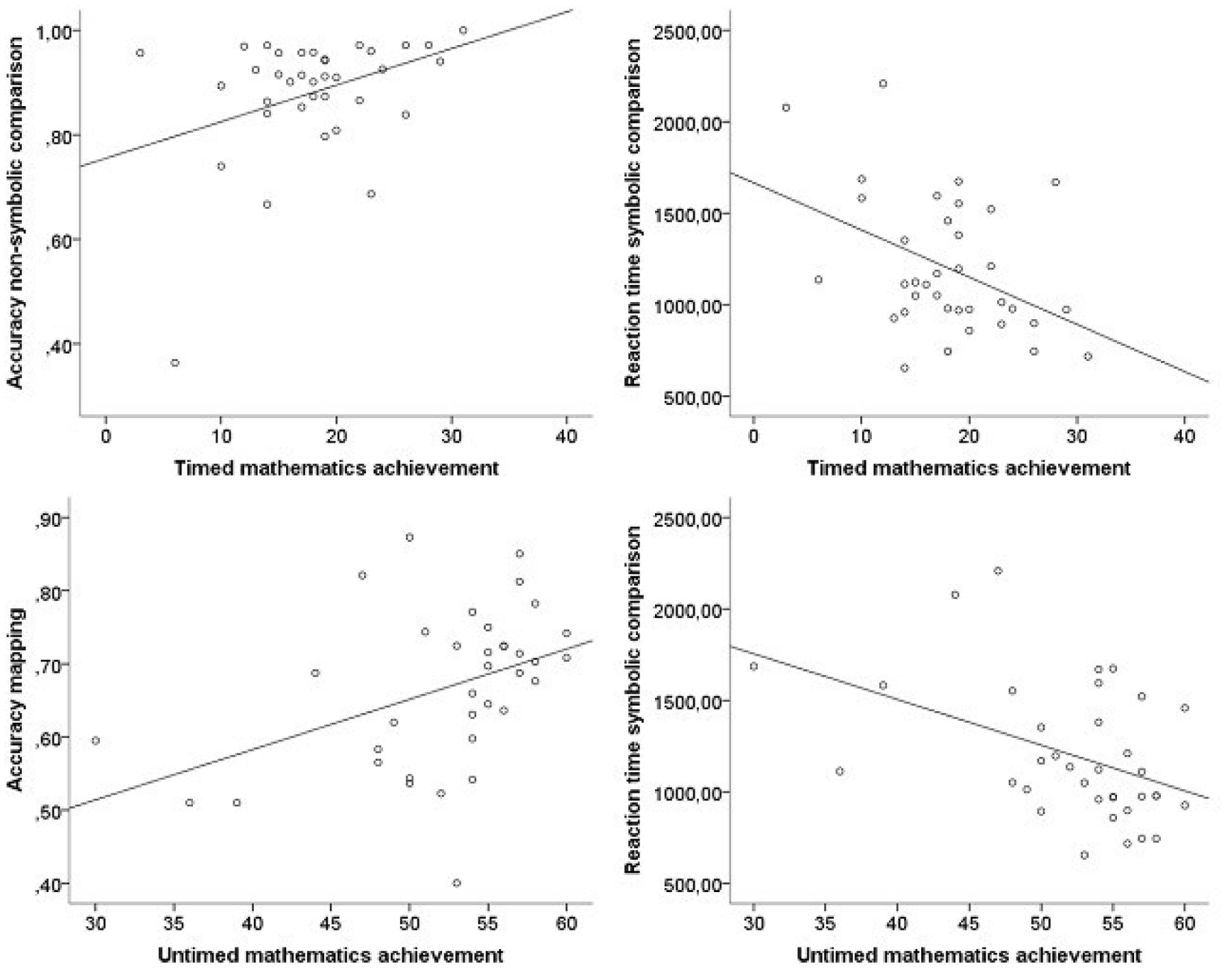
Scatterplots showing the significant associations between the numerical magnitude processing tasks and timed (top panels) and untimed (bottom panels) mathematics achievement in Grade 1.

In *Grade 1*, as shown in Table 2, accuracy on the mapping task showed a significant association with untimed mathematics achievement, indicating that children who were more accurate on this mapping task had higher performance on the untimed mathematics achievement test. Furthermore, accuracy on the non-symbolic magnitude comparison task was significantly associated with timed mathematics achievement, indicating that children who were more accurate on this task solved more arithmetic problems within one minute. Children’s reaction time on the symbolic magnitude comparison task was significantly associated with both timed and untimed tests of mathematics achievement. Scatterplots displaying these associations are presented in Figure 5.

**Figure 6:**
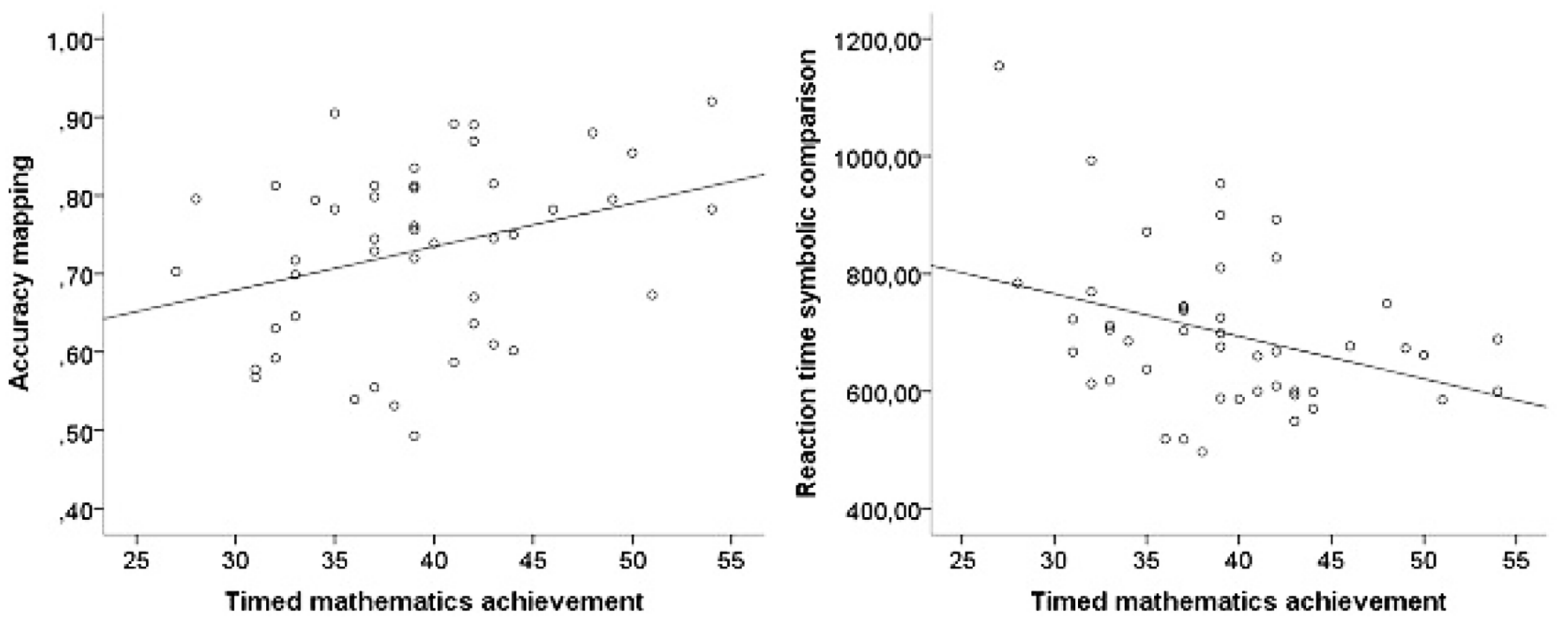
Scatterplots showing the significant associations between the numerical magnitude processing tasks and timed mathematics achievement in Grade 3.

In *Grade 3*, children’s accuracy on the mapping task was significantly correlated with timed mathematics achievement, indicating that children who were more accurate on the mapping task solved more arithmetic problems within one minute. Furthermore, reaction time on the symbolic magnitude comparison task was also significantly associated with timed mathematics achievement: Children who were faster on the symbolic magnitude comparison task were also faster on the timed mathematics achievement test. These associations are displayed in scatterplots in Figure 6. No significant correlations between numerical magnitude processing and untimed mathematics achievement were found.

### 3.3 Regression Analyses

Regression analyses were carried out to further examine the association among our mapping tasks, numerical magnitude comparison and mathematics achievement (Table 3). The numerical tasks that significantly correlated with the mathematics achievement measures, i.e. mapping accuracy, non-symbolic magnitude comparison accuracy and symbolic magnitude comparison reaction time, were simultaneously entered in the model. Analyses were carried out separately for the timed and untimed tests of mathematics achievement and they were run for each grade separately.

**Table 3:** Regression analyses predicting timed and untimed mathematics achievement in Grade 1 and Grade 3.

		Grade 1			Grade 3		
Dependent variable	Predictor	*β*	*t*	*R^2^*	*β*	*t*	*R^2^*
Timed mathematics achievement	Accuracy mapping	.06	0.34	.00	.41	2.87**	.13
	Accuracy comparison NS	.34	2.02	.09	.16	1.07	.02
	Reaction time comparison S	−.43	−2.94**	.19	−.54	−3.92**	.24
Untimed mathematics achievement	Accuracy mapping	.50	3.19**	.19	.08	.49	.01
	Accuracy comparison NS	−.09	−.58	.01	−.10	−.59	.01
	Reaction time comparison S	−.48	−3.48**	.23	−.25	−1.51	.05

NS = non-symbolic; S = symbolic. ** *p<*.01. Both regression models for timed mathematics achievement are significant (Grade 1: *F*(3,32) = 4.89, *p*<.01, *R^2^* = .31; Grade 3: *F*(3,42) = 7.21, *p*<.01, *R^2^* = .34). Regression model for untimed mathematics achievement is significant in Grade 1 (*F*(3,32) = 7.23, *p*<.01; *R^2^* = .40) but not in Grade 3 (*F*(3,42) = 1.15, *p = *.34, *R^2^* = .08).

For timed mathematics achievement in Grade 1, reaction time on the symbolic magnitude comparison task accounted for a significant proportion of variance in math performance, while only a trend towards significance was observed for accuracy on the non-symbolic magnitude comparison task (*p* = .05). Accuracy on the mapping task was not a significant predictor of children’s timed mathematics achievement. After repeating the regression analysis with children’s intelligence and motor reaction time as control variables, reaction time on the symbolic magnitude comparison task failed to reach conventional levels of statistical significance (*p* = .12). Follow-up regression analyses in which motor reaction time or intelligence were considered separately as control variables revealed that this latter finding was due to the control of motor reaction time.

For untimed mathematics achievement in Grade 1, reaction time on the symbolic magnitude comparison task and accuracy on the mapping task accounted for a significant amount of unique variance in math performance, while non-symbolic magnitude comparison did not. After controlling for intelligence and motor reaction time, only symbolic magnitude comparison reaction time remained a significant predictor of untimed mathematics achievement (*p* = .03), while mapping was only marginally related to mathematics achievement (*p* = .07). Follow-up regression analyses in which motor reaction time or intelligence were considered separately as control variables revealed that this latter observation was due to the control of intelligence.

Turning to Grade 3, both accuracy on the mapping task and reaction time on the symbolic magnitude comparison task accounted for a significant proportion of variance in timed math performance, while accuracy on the non-symbolic magnitude comparison task did not. After controlling for intelligence and motor reaction time, both mapping (*p* = .02) and symbolic magnitude comparison (*p*<.01) remained significant predictors of timed mathematics achievement. Because none of the numerical processing measures was significantly correlated with untimed mathematics achievement, it is not surprisingly that no significant associations were found in the regression analyses for untimed math performance.

## Discussion

The ability to map between non-symbolic and symbolic magnitude representations has been proposed to play an important role in the development of mathematics but most of the existing work assessed this ability indirectly by looking at participants’ performance on a symbolic magnitude comparison task [4]–[5], which only implies the direct processing of symbolic stimuli. Some studies have tried to examine mapping more directly by using a mapping task in which participants had to explicitly make connections between symbolic and non-symbolic magnitudes [1], [3], yet their work focused only on quantities larger than or equal to 20. Because Sullivan and Barner [6] argued that different mapping mechanisms operate for linking large (>20) vs. small (≤ 12) non-symbolic and symbolic quantities, the present study extended the existing body of data by investigating for the first time mapping abilities with small quantities ranging from 1 to 9. More specifically, we examined the mapping ability of first and third graders directly by two mapping tasks in which children had to choose which of two quantities (Arabic digits or dot arrays) matched the target quantity (dot array or Arabic digit). We investigated how this mapping ability changed over time, how it was related to timed and untimed math achievement and how these associations between mapping ability and math achievement differed between first and third graders. The present findings indicate that children are able to map between non-symbolic and symbolic representations, that their mapping ability develops over time and that it is related to timed and untimed measures of mathematics achievement.

In the present study, children performed the mapping tasks well above chance and third graders were more accurately than first graders on both the symbolic to non-symbolic and non-symbolic to symbolic mapping task. These findings are in line with Mundy and Gilmore [3], who found a development in children’s mapping ability between 6 and 8 years of age. Moreover, the present findings go beyond the previous ones by showing that this development can also be observed for small quantities. The observed developmental decrease in reaction time, however, could be explained by developmental differences in general response speed. Thus, the specific developmental change in mapping ability seems to be restricted to accuracy. Different from Mundy and Gilmore [3], no asymmetry in children’s mapping direction was found, i.e. they performed equally well on the symbolic to non-symbolic mapping task as on the non-symbolic to symbolic mapping task. This difference in findings might be explained by the fact that smaller quantities were used in the present study. More specifically, the non-symbolic representations associated with smaller quantities were less approximate, leading to a reduction in the disadvantage of dealing with two approximate representations in the symbolic to non-symbolic mapping task compared to one approximate representation in the non-symbolic to symbolic mapping task. On the other hand, these differences in findings could also be explained by differences in sample between the current study and Mundy and Gilmore [3]. Future studies should therefore run mapping tasks with small as well as larger quantities in one sample of children in order to directly investigate how mapping develops over time, and whether there are any differences between the mapping of small and large quantities.

Extending Castronovo and Göbel [1] and Mundy and Gilmore [3], we examined the relationship between children’s numerical magnitude processing skills and their mathematics performance by means of two different types of mathematics achievement tests (timed vs. untimed). This was done because both types of tests are assumed to measure genetically different aspects of math performance [27]. In line with previous studies [3], [4], [13], [15], we observed associations between children’s magnitude comparison skills and their timed and untimed mathematics performance. Moreover, consistent with the literature [4], [15], especially children’s reaction time on the symbolic magnitude comparison task was related to their timed and untimed mathematics achievement. These data provide indirect evidence for the importance of children’s ability to map between Arabic digits and the quantities they represent for their mathematical development. In Grade 1, however, we also observed a correlation between children’s accuracy on the non-symbolic magnitude comparison task and their timed arithmetic performance. This is in line with studies that observed an association between non-symbolic comparison performance and mathematical abilities (e.g., [3], [14]), although there also exist many studies that failed to find such a relationship (e.g., [4], [15], [18]). Differences in task design (e.g. different types of visual control for non-numerical parameters) and performance measures on the non-symbolic tasks (e.g. using accuracy or Weber fractions) probably explain these inconsistencies (see [11], [34] for a detailed discussion).

Because the numerical magnitude comparison tasks only provide an indirect measure of mapping skills, we also calculated the correlations between children’s performance on the mapping tasks and the timed vs. untimed mathematics achievement test. Extending the results of Mundy and Gilmore [3], different associations between mapping and mathematics performance were found depending on grade and the type of mathematics achievement test that was used. In Grade 1, children’s mapping ability was related to untimed mathematics achievement, while in Grade 3 an association was found between mapping and a timed arithmetic test.

Finally, similar to the findings of Mundy and Gilmore [3], we found that children’s mapping ability was related to their timed (Grade 3) and untimed (Grade 1) mathematics performance over and above the variance accounted for by numerical magnitude comparison skills. Extending previous work [1], [3], these findings seem to suggest that also the ability to map directly between small (1–9) non-symbolic and symbolic magnitude representations plays an important role in the development of mathematics, and that this ability cannot be fully captured by looking at symbolic comparison performance alone.

The present findings also shed a new light on the assumption of Lyons, Ansari and Beilock [35] that numerical symbols operate primarily as an associative system that is distinct from non-symbolic magnitude representations. The fact that children’s mapping ability was uniquely related to mathematics performance suggests that the association between numerical symbols and their corresponding non-symbolic representations is important for mathematical development, at least at a relatively young age. It might well be, as suggested by Lyons et al. [35], that the relations between symbols come to overshadow the relation between symbolic and non-symbolic magnitudes at later ages, yet longitudinal research across a very wide age range is needed to test this hypothesis.

The current findings demonstrate that children’s ability to map between non-symbolic and symbolic representations is uniquely related to their timed and untimed mathematical abilities, although future studies are needed to determine the causal direction of this relationship, i.e. to determine whether children’s mapping ability predicts, or is itself enhanced by, their mathematical abilities. If the ability to map between non-symbolic numerical magnitudes and Arabic digits underlies children’s mathematical development, it may be an important target for teaching and remediation of children with mathematical difficulties.

## References

[1] Castronovo J, Göbel SM (2012) Impact of high mathematics education on the number sense. PloS One 7: e33832. 22558077 10.1371/journal.pone.0033832 PMC3338810

[2] Mazzocco MMM, Feigenson L, Halberda J (2011) Impaired acuity of the approximate number system underlies mathematical learning disability (dyscalculia). Child Dev 82: 1224–1237. 21679173 10.1111/j.1467-8624.2011.01608.x PMC4411632

[3] Mundy E, Gilmore CK (2009) Children’s mapping between symbolic and nonsymbolic representations of number. J Exp Child Psychol 103: 490–502. 19327782 10.1016/j.jecp.2009.02.003

[4] Holloway ID, Ansari D (2009) Mapping numerical magnitudes onto symbols: The numerical distance effect and individual differences in children’s mathematics achievement. J Exp Child Psychol 103: 17–29. 18513738 10.1016/j.jecp.2008.04.001

[5] Rousselle L, Noël MP (2007) Basic numerical skills in children with mathematics learning disabilities: a comparison of symbolic vs. non-symbolic number magnitude processing. Cognition 102: 361–395. 16488405 10.1016/j.cognition.2006.01.005

[6] Sullivan J, Barner D (2013) How are number words mapped to approximate magnitudes? Q J Exp Psychol 66: 389–402. 10.1080/17470218.2012.715655 22963174

[7] Dehaene S (1997) The number sense: how the mind creates mathematics. New York: Oxford University Press.

[8] Xu F, Arriaga RI (2007) Number discrimination in 10-month-old infants. Br J Dev Psychol 25: 103–108.

[9] Barth H, Beckmann L, Spelke ES (2008) Nonsymbolic, approximate arithmetic in children: Abstract addition prior to instruction. Dev Psychol 44: 1466–1477. 18793077 10.1037/a0013046 PMC3489021

[10] Griffin S (2003) The development of math competence in the preschool and early school years: cognitive foundations and instructional strategies. In: Royer JM, editor. Mathematical Cognition. Connecticut: Information Age Publishing. 1–32.

[11] De Smedt B, Noël MP, Gilmore C, Ansari D (2013) The relationship between symbolic and non-symbolic numerical magnitude processing and the typical and atypical development of mathematics: evidence from brain and behavior. Trends Neurosci Educ 2: 48–55.

[12] Bugden S, Ansari D (2011) Individual differences in children’s mathematical competence are related to the intentional but not automatic processing of Arabic numerals. Cognition 118: 35–47. 10.1016/j.cognition.2010.09.005 20970782

[13] De Smedt B, Verschaffel L, Ghesquière P (2009) The predictive value of numerical magnitude comparison for individual differences in mathematics achievement. J Exp Child Psychol 103: 469–479. 19285682 10.1016/j.jecp.2009.01.010

[14] Mazzocco MMM, Feigenson L, Halberda J (2011) Preschoolers’ precision of the approximate number system predicts later school mathematics performance. PloS One 6: e23749. 21935362 10.1371/journal.pone.0023749 PMC3173357

[15] Sasanguie D, Göbel SM, Moll K, Smets K, Reynvoet B (2013) Approximate number sense, symbolic number processing, or number-space mappings: What underlies mathematics achievement? J Exp Child Psychol 114: 418–431. 23270796 10.1016/j.jecp.2012.10.012

[16] Kolkman ME, Kroesbergen EH, Leseman PPM (2013) Early numerical development and the role of non-symbolic and symbolic skills. Learn Instr 25: 95–103.

[17] Halberda J, Mazzocco MMM, Feigenson L (2008) Individual differences in non-verbal number acuity correlate with maths achievement. Nature 455: 665–668. 18776888 10.1038/nature07246

[18] De Smedt B, Gilmore CK (2011) Defective number module or impaired access? Numerical magnitude processing in first graders with mathematical difficulties. J Exp Child Psychol 108: 278–292. 20974477 10.1016/j.jecp.2010.09.003

[19] Landerl K, Bevan A, Butterworth B (2004) Developmental dyscalculia and basic numerical capacities: A study of 8–9-year-old students. Cognition 93: 99–125. 15147931 10.1016/j.cognition.2003.11.004

[20] Mussolin C, De Volder A, Grandin C, Schlögel X, Nassogne MC, et al (2010) Neural correlates of symbolic number comparison in developmental dyscalculia. J Cogn Neurosci 22(5): 860–874. 19366284 10.1162/jocn.2009.21237

[21] Butterworth B, Varma S, Laurillard D (2011) Dyscalculia: From brain to education. Science 332: 1049–1053. 21617068 10.1126/science.1201536

[22] Mussolin C, Mejias S, Noël MP (2010) Symbolic and non-symbolic number comparison in children with and without dyscalculia. Cognition 115: 10–25. 20149355 10.1016/j.cognition.2009.10.006

[23] Noël MP, Rousselle L (2011) Developmental changes in the profiles of dyscalculia: an explanation based on a double exact-and-approximate number representation model. Front Hum Neurosci 5: 165. 22203797 10.3389/fnhum.2011.00165 PMC3243900

[24] Lipton JS, Spelke ES (2005) Preschool children’s mapping of number words to nonsymbolic numerosities. Child Dev 76: 978–988. 16149996 10.1111/j.1467-8624.2005.00891.x

[25] Barth H, Starr A, Sullivan J (2009) Children’s mappings of large number words to numerosities. Cognitive Dev 24: 248–264.

[26] Izard V, Dehaene S (2008) Calibrating the mental number line. Cognition 106: 1221–1247. 17678639 10.1016/j.cognition.2007.06.004

[27] Petrill S, Logan J, Hart S, Vincent P, Thompson L, et al (2012) Math fluency is etiologically distinct from untimed math performance: evidence from a twin study. J Learn Disabil 45: 371–381. 21890908 10.1177/0022219411407926 PMC3413280

[28] Raven JC, Court JH, Raven J (1992) Standard progressive matrices. Oxford, UK: Oxford Psychologists Press.

[29] Schneider W, Eschmann A, Zuccolotto A (2002) E-prime reference guide. Pittsburg, PA: Psychology Software Tools.

[30] Pica P, Lemer C, Izard W, Dehaene S (2004) Exact and approximate arithmetic in an Amazonian indigene group. Science 306: 499–503. 15486303 10.1126/science.1102085

[31] Sekuler R, Mierkiewicz D (1977) Children’s judgments of numerical inequality. Child Dev 48: 630–633.

[32] De Vos T (1992) Tempo-Test-Rekenen. Handleiding. [Tempo Test Arithmetic. Manual]. Nijmegen: Berkhout.

[33] Dudal P (2000) Leerlingvolgsysteem: Wiskunde–Toetsen 1–2–3 Basisboek [Student monitoring system: Mathematics–Tests 1–2–3 manual]. Leuven, Belgium: Garant.

[34] Inglis M, Gilmore C (2014) Indexing the approximate number system. Acta Psychol 145: 147–155. 10.1016/j.actpsy.2013.11.009 24361686

[35] Lyons IM, Ansari D, Beilock SL (2012) Symbolic estrangement: evidence against a strong association between numerical symbols and the quantities they represent. J Exp Psychol: General 141: 635–641. 10.1037/a0027248 22329752

